# Introduction of Formative Assessment Classroom Techniques (FACTs) to School Chemistry Teaching: Teachers’ Attitudes, Beliefs, and Experiences

**DOI:** 10.1021/acs.jchemed.3c00591

**Published:** 2023-08-25

**Authors:** Mária Babinčáková, Mária Ganajová, Paweł Bernard

**Affiliations:** † Pavol Jozef Šafárik University in Košice, Lifelong Learning Centre and Project Support, Šrobárova 2, Košice 041 80, Slovakia; ‡ Pavol Jozef Šafárik University in Košice, Faculty of Science, Department of Didactics of Chemistry, Šrobarová 2, Košice 041 80, Slovakia; § Jagiellonian University, Faculty of Chemistry, Department of Chemical Education, Gronostajowa Str. 2, Kraków 30-387, Poland

**Keywords:** Middle School Science, Continuing Education, Curriculum, Testing/Assessment, Chemical Education Research

## Abstract

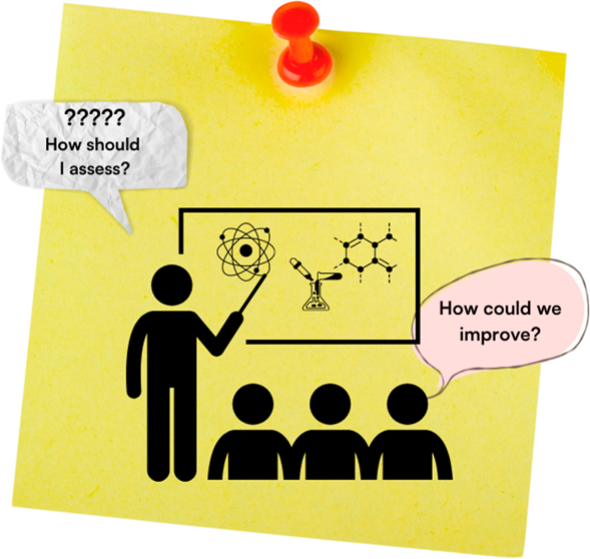

This paper presents teachers’ perspectives and experiences with the implementation of formative assessment (FA) into chemistry lessons at the secondary school level through Formative Assessment Classroom Techniques (FACTs). The research had a qualitative character and was based on semistructured interviews focused on: the definition and previous use of FA, implementation experience, and teachers’ beliefs, attitudes, and abilities. The research describes five cases—chemistry teachers participating in a professional development program. The 2 year-long training was focused on the theory of FA, practical exercises, and extended support during in-school FACTs implementation. The results showed that using FACTs during secondary school chemistry lessons emphasizes students’ strengths and weaknesses, encourages them to perform truthful self-assessments, and engages them. Moreover, using FACTs opens new areas for parents’ involvement in the assessment and learning process that can be especially valuable for students with special educational needs. The main challenges cited by teachers were time management, policy support, and the need for further assistance during FACTs implementation.

## Introduction

In many countries, education at the preschool and primary levels (K1–4) is based on a constructivist paradigm, natural cognition of the world, and surrounding phenomena. This process is assisted by teachers who use formative assessment (FA) to guide students and inform them and their parents about the progress they make. Unfortunately, moving to upper levels of education, teaching and assessment methods are changing. Middle school (K5–9) and secondary school (K10–12) levels bring formalization of the educational process and almost a complete shift toward summative assessment.^1^ Primary school teachers (K1–4) are usually able to use various methods and types of assessment that employ various types of evidence. Teachers at the primary school level clearly use a more pedagogically oriented (process-oriented) assessment, while teachers at the secondary school level focus on subject-specific knowledge (goal-oriented approach). However, recently, educational policy in many countries has changed and forced, or at least advised, using a formative assessment at the secondary school level and in higher education.^2−7^

Formative assessment, also called assessment for learning, identifies the individual learning needs of students and provides tailored instruction to meet them.^8^ Such feedback must be provided at the point where students still have the opportunity to use it and improve their learning.^9,10^ Black and Wiliam^11^ stated that *“formative assessment can be conceptualised of five key strategies*:

(1) *clarifying and sharing learning intentions and criteria for success;*
(2) *engineering effective classroom discussions and other learning tasks that elicit evidence of students’ understanding;*
(3) *providing feedback that moves learners forward;*
(4) *activating students as instructional resources for one another; and*
(5) *activating students as the owners of their own learning”* (p. 4).

As was pointed out by Hattie and Timperley, even though feedback is a very powerful tool, we always must think about its positive and negative effects.^12^ Constructive feedback requires much more skilled students and teachers, and its inappropriate use could bring more harm than positive results.^12^

### Formative Assessment Classroom Techniques

The methodology of formative assessment has been developing over the years, and various models, ideas, and practices have been used to support students’ learning.^13−15^ However, the key points remain similar; formative assessment should provide to students a target to reach, a point where they are, and the way to close the gap.^16^ Such feedback can be delivered via “Formative Assessment Classroom Techniques” (FACTs),^17^ which are quick tools that provide information to a student and a teacher instantly. Among the FACTs, we can feature checklist,^18,19^ concept map,^20−22^ exit card,^23−25^ K-W-L chart,^26^ true or false statements,^4,27^ or vocabulary square (Frayer model).^28,29^ Brief descriptions and the main characteristics of each are presented in Table 1. Each provides personalized, spontaneous feedback facilitating learning, and their completion does not take long.^29,30^ FACTs were found to be useful in teaching foreign languages, literature, and art and, lately, also have been applied to science and chemistry education. Its effectiveness was studied mainly at the primary and tertiary levels,^31,32^ and it has been proven that using FACTs improves students’ learning outcomes,^6^ increases their motivation,^31^ and has a positive impact on teachers’ professional development.^32^

**Table 1:** Detailed Description of Formative Assessment Classroom Techniques (FACTs)

	Description	
FACT	How Is It Done?	What Is It Used For?
checklist	Completion of the task is rated according to a chosen scale, e.g.: 5-point (1 = poor, 5 = excellent), 3-point (I can do it alone, I can do it with little help, I cannot do it at all), and 2-point (yes, no). The assessment can be performed by a teacher, by a classmate (peer assessment), by a student him or herself (self-assessment), or by a combination of those.^19,32−35^	• reflection on student’s knowledge
		• guidance for student’s knowledge
		• identifying the presence or absence of knowledge
		• identifying completion of tasks
concept map	Concept map can be assessed by the number of used words and connections (relationships) made between them. Analysis of the connections leads to identifying students’ knowledge and understanding relations within the topic. It can be used at the beginning of the lesson to facilitate the discussion or at the end to wrap up the topic.^22,36^	• identification of student’s ideas and understanding of the topic
		• organization of student’s knowledge
		• visualization of student’s knowledge
		• forming relations and networks between issues
		• helps with memorizing and organizing FACTs
exit card	Additionally called a minute paper, 1 min paper, or 3–2–1 card. It is usually used at the end of the lesson. We ask our students about three key points from the lesson, two interesting facts, and one unanswered question. It is important that students’ questions be clarified.^23−25,37^	• feedback on student’s understanding of the topic
		• identifying students who need more or special assistance
K-W-L	At the beginning of the lesson, students write what they know (K) about the topic and what they would like to know (W) about it. At the end of the lesson, students write what they have learned (L).^38^	• review of prior knowledge
		• organization of ideas
		• development of critical thinking
true or false statements	This tool is based on various statements about the topic. At the beginning of the lesson, students decide whether the statements are true or false. At the end of the lesson, they judge the same statements again. They can compare and discuss their entry decisions with the final ones.^4^	• activating student’s thinking about the particular topic
		• reflecting on the lesson
vocabulary square	Also called Frayer model. Around the word or phrase, students draw four blocks. In the first block, they write the definition of the word (phrase), in the second one the facts or characteristics of it, in the third one examples, and in the last one nonexamples.^28^	• organization of information
		• categorization of information
		• supporting the vocabulary
		• supporting concept thinking

### Formative Assessment in Chemistry Education

Even though the topic of formative assessment is considered as widely known and used, there are very few examples of using this kind of assessment in chemistry education at the secondary school level and even fewer examples of teachers’ practices. One study presents the computer model-based formative assessment tests to measure high school students’ chemical reasoning, although no feedback from students or teachers is presented.^39^ Feedback focused on the correctness of students’ responses was used after online chemistry activities in a virtual lab.^40^ Vogelzang and Admiraal added formative assessment to context-based chemistry teaching and statistically proved the positive effect of this kind of assessment on students’ achievement.^41^ One part of the OCIA! program developed as an alternative mode of teaching organic chemistry was using various types of formative assessment (class discussions, worksheets, card-game activities, group classroom assessments, etc.) during organic chemistry lessons.^42^ Using formative assessment during science camp was also documented.^43^ Easa and Blonder developed customized pedagogical kits to help chemistry teachers personalize their teaching according to students’ misconceptions.^44^ As is visible, the research on the implementation of FA into chemistry education is more focused on the implementation of formative assessment or feedback rather than FACTs. Early research describing using FA in chemistry education appeared only during the past decade and mostly in the United States and Western Europe.^39−42^ Meanwhile, using this kind of assessment in the rest of the world is slowly beginning to emerge.^6,43,44^

### Teachers’ Training

Black and Wiliam^10^ noticed that changing teachers’ assessment strategies requires time. They stated, *“There is no “quick fix” that can be added to existing practice with promise of rapid reward...each teacher finds his or her own ways of incorporating the lessons and ideas that are set out above into his or her own patterns of classroom work. This can only happen relatively slowly, and through sustained programmes of professional development and support.”* In fact, the design of the teacher’s professional development program within the project promoting and incorporating FA is the major challenge. Lee and William analyzed this problem in detail.^45^ They identified six aspects of the project’s strategy supporting teacher changes:

(1) credible evidence presented to teachers,
(2) practical ideas offered to them,
(3) meetings in which they could give one another mutual support,
(4) supportive environment for exchanges of details and reflection about their actions,
(5) time, in Lee and Wiliam’s project at the end of the first year, there were only modest changes in the teachers’ actual classroom practices, and radical changes started to appear during the second year, and
(6) flexibility, teachers should be encouraged to make their own choices and explorations from the menu of possible changes.

These aspects lie in line with the current understanding of the role and shape of teachers’ professional development programs.^46^ Liberman noticed that the ways teachers learn may be more similar to the way students learn than was previously recognized.^46^ Teachers learn by doing, reflecting, collaborating with peers, analyzing students’ work, and discussing results.^47^ In all this, teachers beliefs play a key role. *“Teacher beliefs are implicit and explicit suppositions held by educators which have relevance for their professional and instructional practices, interactions with students, and learning processes. They may include beliefs about students, self, learning, knowledge, and knowing.”*(48) Moreover, Harrison, Howard, and Matthews^49^ claim that teachers need to experience every new method as learners themselves because durable changes in pedagogy occur only when the teachers understand how their students feel in their classroom. Finally, the opportunity for cooperation with peers is crucial to understand what is expected from them, what changes they might make in their practice, and what effects they can expect.^49−53^ Therefore, professional development programs should contain work in groups not only during the main training but also while following experiences with multiple opportunities for interaction.^54^

## Research Background

### Formative Assessment in Slovakia

The term *“formative assessment”* (in Slovak *“formatívne hodnotenie”*) was first mentioned in Slovak papers in the 2000s and was focused on assessing preservice teachers during their training.^55^ Afterward, the enhancement of formative assessment was related to the introduction of inquiry-based methods^56,57^ and the realization of the two European FP7 projects: ESTABLISH (2010–2014)^58^ and SAILS (2012–2015).^59^ Currently, there are several national projects that focus on the implementation of formative assessment in various school subjects, and this topic is spread across the country.^6,60−63^ Orosová et al.^64^ studied the status of assessment in Slovak schools in STEM. She concluded that teachers prefer using summative assessment over formative assessment. A similar conclusion was presented in the OECD Reviews of Evaluation and Assessment in Education: Slovak Republic:^65^*“Summative assessment plays a strong role in Slovak primary and secondary schools...The predominant culture appeared rather to be the use of regular and frequent summative assessment (such as weekly quizzes followed by more formal tests at the end of a topic) with the main purpose to provide evidence towards students’ grades at the end of each semester.”* Therefore, there is a need for teacher training, popularization, and development of FA in Slovakia.

### The Project

The presented research was based on the project titled “IT Academy” run in Slovakia in 2016–2022, devoted to teacher training in new trends in chemistry education.^66,67^ One of the profiles of teacher development was the implementation of formative assessment in chemistry lessons. The core training and sustained support were designed to meet the recommendations made by Lee and Wiliam.^45^ The training was voluntary and free of charge, and admission was on a “first come first serve” basis. After the training, teachers received a certificate of attendance. Participants were expected to attend at least 80% of the meetings. The training started in the summer of 2018 with theoretical classes where teachers learned about current trends in teaching chemistry (e.g., inquiry-based science education, project-based learning, and teaching chemistry with digital technologies), differences between formative and summative assessment, the history and development of formative assessment, and different approaches to FA, including the idea and examples of FACTs. Subsequently, teachers moved to practical training, and they tried to prepare their own FACTs for various chemistry topics. They discussed the pros and cons of particular FACTs, the possibility of embedding them into syllabi, and the topics appropriate for trial implementation, and they drafted FACTs for particular lessons. Draft FACTs and lesson scenarios were polished by tutors and delivered in the form of complete, ready-to-use scenarios.^6,60^ These measures were implemented by teachers at the beginning of the 2018/2019 school year (September–December 2018). During implementation, tutors were in touch with teachers providing necessary support. Moreover, tutors attended the chosen lessons of each teacher to see and discuss her or his practices. After the implementation stage, several group meetings were organized to create opportunities for exchanging experiences (spring and summer 2019). Additionally, during these meetings, the results of the implementation were presented and discussed so that teachers could see how using FACTs influenced their students’ skills and knowledge.^6^ After these meetings, during the 2019/2020 school year (September 2019–February 2020), teachers participated in the second round of the implementation process. This time, teachers were not obligated to use the provided scenarios, and the form and frequency of using FACTs/FA were to be decided by the teachers. After the implementation process, individual interviews with teachers were conducted. One of the purposes of the interviews was to help teachers analyze the process, reflect on FACTs usage, and verbalize the pros and cons of FA realized via FACTs. The schedule and range of the entire training program are presented in Table 2.

**Table 2:** Teachers’ Training Outline

Type of Support	Type of Meeting	Duration	Period
Introduction to FA, theoretical background	F2F	2 h	summer 2018
Design and development of FACTs with tutors	F2F	4 h	summer 2018
Own development of FACTs	Distance-Asynchronous	2 h	summer 2018
Preparation of classroom implementation stage 1	F2F	2 h	September 2018
FACTs implementation stage 1 (based on scenarios designed with peers and approved by tutors) *(lectors attended a few lessons)*		10 lessons	September 2018–December 2018
Evaluation of implementation stage 1	F2F	3 × 1.5 h	spring and summer 2019
Preparation of classroom implementation stage 2	F2F	1 h	September 2019
FACTs implementation stage 2 (based on own designed scenarios) *(without presence of lectors)*		approximately 20 lessons	September 2019–February 2020
Evaluation of implementation stage 2 and interviews	Distance-Synchronous	1 h (online)	spring 2020

The second stage of FACTs implementation was also designed to meet Lee and Wiliam^45^ recommendations; therefore, teachers practiced FACTs implementation in a real school environment, and reliable evidence of how FACTs implementation influenced their student’s outcomes and attitudes was presented. The meetings were organized such that teachers could exchange experiences, were provided with a supportive environment, had long-term experience since both stages of implementation lasted almost 24 months, and had flexibility in how and the frequency with which the method was used. The evidence of how using FACTs influenced students’ outcomes gathered during the first stage of FACTs implementation was described by Babinčáková et al.^6^ The results suggested a positive impact of FACTs on students’ outcomes and positive attitudes toward the approach.

## Research Questions

The research focuses on the usefulness and implementation process of FACTs while teaching chemistry at the secondary school level. The chosen research questions are as follows:

- How have the chemistry teachers’ beliefs and motivations been influenced by the series of training and implementations of FA/FACTs?
- What are chemistry teachers’ experiences with the implementation of FA based on FACTs?
- How has long-time training influenced chemistry teachers’ confidence in using FA and FACTs?

## Methodology

### Research Design

The case study was run during the spring of 2020, during the second FACTs implementation by teachers. Teachers have been introducing FA based on FACTs from the beginning of the 2019/2020 school year for freshman secondary school students (K7 level) in chemistry classes. At this level, students in Slovakia meet chemistry as a separate subject for the first time. At this stage of the project (training), teachers could decide independently about the number of FACTs used, their role, and form. Topics taught during this period were determined by curriculum and included mixtures, water, and air.

Analysis of the FACTs implementation process in school everyday practice requires qualitative analysis to identify and trace teachers’ practices, skills, plans, problems, beliefs, and attitudes.^68,69^ Therefore, the case study was based on extensive and structured interviews conducted after 6 months of stage 2 FACTs implementation.

### Participants

The teachers who participated in this study were chemistry teachers realizing the professional development program focused on the implementation of formative assessment within the “IT Academy” project.^6,60^ All of them finished the first and second stages of the course. Participation in the study, as in the whole project, was voluntary and could be abandoned at any stage of the project. Twenty-eight teachers participated in the whole project, and all of them were asked to participate in the research; 5 answered positively. Therefore, those teachers were highly motivated and participated enthusiastically in the following stages of the training and the research. All the teachers who participated in the project were women, which reflects a general gender structure in the teacher profession in Slovakia, in which 80% of Slovak teachers are women.^70^ The nicknames of the teachers, the subjects taught, their experience, and their short characteristics based on the introductory survey and our experience are presented in Table 3.

**Table 3:** Characteristics of Teachers Participating in the Research

Name	Teaching Experience [years]	Subjects Taught	Characteristics
Alice	29	Chemistry, mathematics	A vice-principal of the school. She sees her job as a mission and an opportunity to help students be good people.
Andrea	26	Chemistry, biology	She tries to use modern methods during her lessons. She usually teaches all students in different classes at the same level.
Maria	11	Chemistry	She initially worked in the chemical industry. Her view of a teacher is to be a punctual chemist in every way.
Martina	20	Chemistry, biology	She wants students to like chemistry as much as possible. For that, she tries to use many experiments, innovations, and interactive ways of teaching.
Svetlana	29	Chemistry, mathematics	She likes her way of teaching, but she knows that it is important to implement new methods to keep students’ attention.

### Questionnaire and Data Analysis

The research involved a structured interview based on the questionnaire presented in the Supporting Information. Some questions were adapted from earlier research.^7,71−73^ The questionnaire consisted of five sections with various numbers and types of questions (see Table 4):

(1) Definition and previous use of formative assessment and FACTs.^71^
(2) Experience from FACTs implementation.^7,71,73^
(3) Teachers’ beliefs about FACTs.^7^
(4) Skills gained during FACTs implementation.^7^
(5) Teachers’ attitudes and plans for the future use of FACTs.^7^

**Table 4:** Types and Number of Questions in the Questionnaire

Section	Type of Question	Number of Questions
Definition and previous use of formative assessment and FACTs	Open	1
	Open conditional	2
	Yes-No	1
	Yes-No conditional	1
	Multiple-choice unipolar	1
	Multiple-choice unipolar conditional	1
	**TOTAL**	**7**
Experience from FACTs implementation	Open	9
	Open conditional	8
	Yes-No	1
	Multiple-choice	1
	Multiple-choice bipolar	3
	Multiple-choice bipolar conditional	1
	Multiple-choice unipolar	6
	Multiple-choice unipolar conditional	4
	**TOTAL**	**33**
Teachers’ beliefs about FACTs	Open	4
	Open conditional	2
	Multiple-choice bipolar	13
	Multiple-choice unipolar conditional	1
	**TOTAL**	**20**
Skills gained during FACTs implementation	Multiple-choice bipolar	5
	**TOTAL**	**5**
Teachers’ attitudes and plans for the future use of FACTs	Open	3
	Multiple-choice bipolar	4
	**TOTAL**	**7**

The questionnaire was developed in English and translated into Slovak. To ensure the clarity of translation, two separate versions were translated independently by one of the authors and an independent researcher. Versions were compared and discussed, and a common version was agreed upon. This document was back translated into English by another independent researcher for verification.

Interviews were conducted by one of the authors in March/April 2020, after FACTs’ stage 2 implementation. Due to the COVID-19 pandemic outbreak, interviews were carried out remotely using the BigBlueButton conference environment. The software allowed audio and video communication as well as a recording to be made of the meeting. Questions were not available to the teachers before the interview. Each interview lasted between 40 and 50 min. Afterward, interviews were transcribed, coded, and analyzed. Descriptive answers were translated into English. Additionally, in this case, back translation was used to verify the exactness of the translation. All multiple-choice bipolar and unipolar questions were coded with numbers (for multiple-choice bipolar questions: (1) Definitely not; (2) No; (3) It is hard to say; (4) Yes; (5) Definitely yes; and for multiple-choice unipolar questions: (1) Never; (2) Rarely (once or twice); (3) Occasionally (few times); (4) Often (almost after every lesson); (5) Very often (after every lesson)). The analysis of the gathered data had qualitative characteristics of particular cases with basic statistics for cross-analysis.

The research was performed in compliance with applicable requirements and Pavol Jozef Šafárik University in Košice ethical guidelines. Teachers participated in the study voluntarily; they were informed about the research design and the data to be collected, and they could withdraw from the research or withhold their data at any stage of the research.

## Results

### Definition and Previous Use of Formative Assessment and FACTs

In the first question, teachers were asked to describe FA in their own words. According to Alice, FA gives feedback to a teacher during the teaching process, not at the end. A teacher can work with this feedback, make improvements, or add something to the lesson scenario. For Andrea, FA is a verbal assessment. It is an assessment done not only by a teacher but also by the students themselves. Maria said, *“When I assess my students in a formative way, then I can verbally assess how they handled the curriculum. Obviously, according to my formative assessment, students can improve gaps in their knowledge.”* Martina describes formative assessment as a kind of wordy assessment or feedback for the immediate status of a student’s learning. It is an assessment for themselves, and it provides feedback about their understanding of the topic. This assessment moves students forward in learning. If there are some unclear tasks, a student can work on them. Svetlana sees formative assessment as a kind of effective education based on student-teacher and teacher-student communication. Students should have their own responsibility for their learning, and they should know how they progress in learning. It should mainly help the student during learning.

Only Andrea and Maria declared that they had known the FA definition and had been using FA before the training; Maria had occasionally used FA, and Andrea had done so very often. The other 3 participants did not know the FA definition before the training, even though Alice and Martina claimed to have used it rarely (a few times a year). Only Svetlana declared not having using FA at all before the training.

### Teachers’ Beliefs about FACTs

Teachers participating in the research had a positive attitude toward FACTs (the mean score for all questions presented in Table 5 was 4.17). The most positive was Andrea, and the least positive was Alice. There were no negative answers (“1-definitely not” nor “2-no”). The response “3-it is hard to say” was used 14 times, the response “4-yes” 30 times, and the response “5-definitely yes” 26 times. The most positive answers were for question number 7, “Using FACTs helps students recognize their strengths and weaknesses in their knowledge”, where all teachers answered “5-definitely yes”. The positive responses were for question number 9, “Using FACTs during chemistry lessons influenced students’ learning in other subjects”, with 4 answers, “3-it is hard to say”, and one answer, “4-yes”.

**Table 5:**
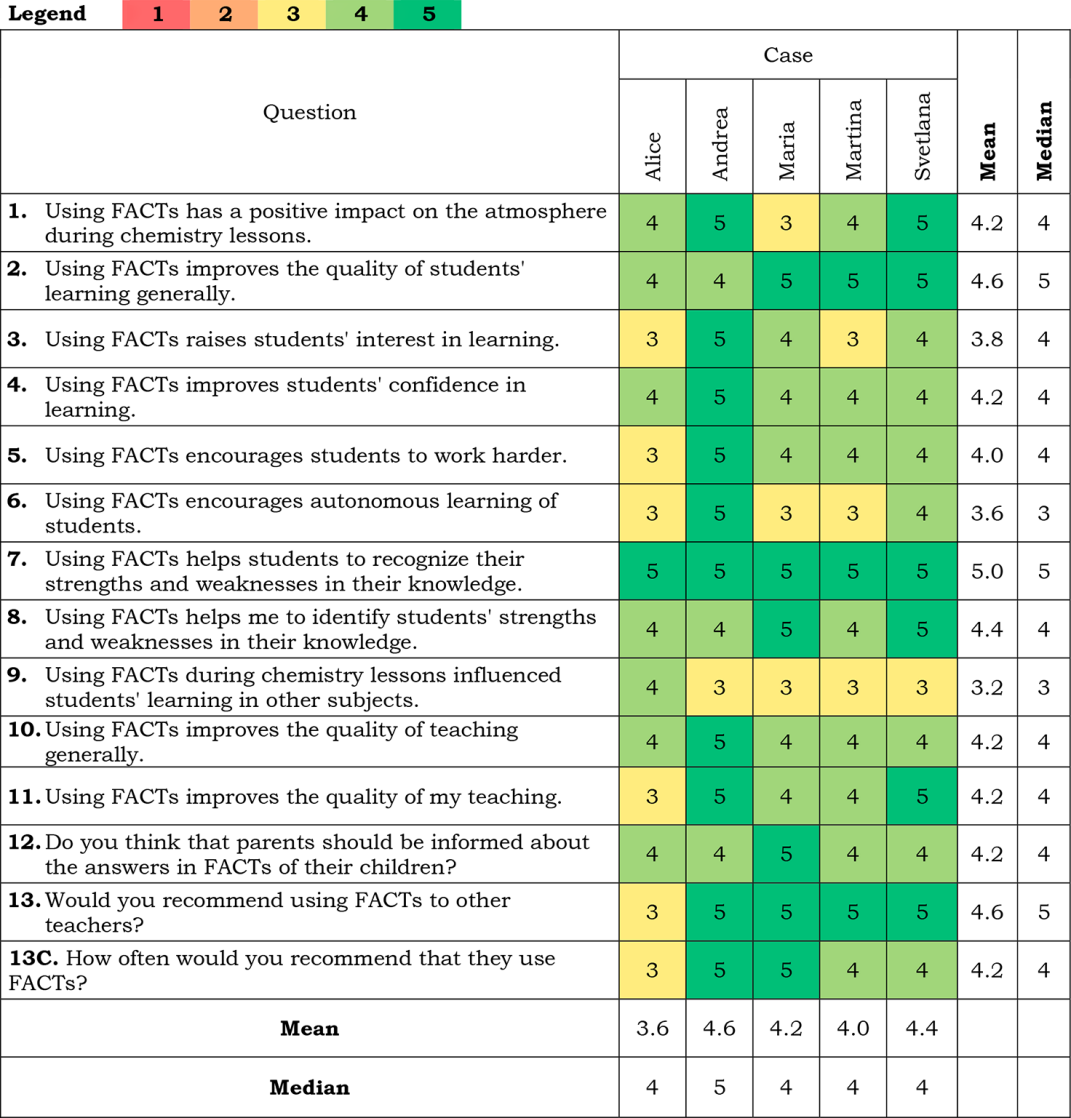
Teachers’ Answers to Questions about Beliefs^a^

a For questions 1–13: (1) Definitely not; (2) No; (3) It’s hard to say; (4) Yes; (5) Definitely yes; for question 13C: (1) Never; (2) Rarely (once or twice); (3) Occasionally (a few times); (4) Often (almost after every lesson); (5) Very often (after every lesson).

Questions 2, 10, 11, 12, and 13 required some descriptive commentary to provide numerical justification of the answer.

Comments to question no. 2 (Using FACTs generally improves the quality of students’ learning) showed that teachers found using FACTs difficult at the beginning. Svetlana explained, “*Well, at the beginning, students have problems with new things.”* Later, when students learned how to use FACTs and they got used to them, their value increased. Working with FACTs helped students focus on their answers, come back, and clarify the wrong ones. On the other hand, teachers were concerned about using FACTs with more difficult and complex topics.

Teachers’ justifications for answers to question no. 10 (Using FACTs generally improves the quality of teaching) underscored that FACTs served as a guide for students’ learning. Students could follow the key points used in a particular FACT. Additionally, FACTs offered a place for everyone in the class to express their opinion or questions about the topic. Alice described it: *“Here you go deeply into learning. It is not focused on a grade, but rather on their knowledge. Maybe they don’t know the definition word by word, but they see relationships. Students are more open, and I can react to their problems.”*

Comments to question no. 11 (Using FACTs improves the quality of my teaching) showed a large gap while using FACTs. This gap was caused by specific problems that occurred during the lesson. Some teachers complained that, when they had difficulties fulfilling the prepared lesson plan, they could not also use the planned FACT, and it disrupted the lesson. On the other hand, teachers noticed that using FACTs helped them identify problems and misunderstandings before a test, so they could return to the topic and discuss it again.

Involving parents in the formative assessment process offered a new view of FACTs. In comments to question no. 12 (Do you think that parents should be informed about the answers in FACTs of their children?), teachers suggested that FACTs could be very useful in helping students with special educational needs and their teaching assistants to identify problematic issues. Accordingly, they could focus on those elements. Furthermore, FACTs were very helpful as a justification for students’ grades. Finally, teachers mentioned that parents cannot be forced to use and work with FA but that teachers could encourage it. Alice said, *“At the beginning of the academic year, we could tell parents that there is a possibility of this feedback, and they could choose using it or not.”*

The recommendation of using FACTs was discussed in the comments to question no. 13 (Would you recommend using FACTs to other teachers? If Yes: What would you say to them?).

Teachers’ positive attitudes toward using FACTs were clearly visible here, but they also underlined the effort required for using FACTs in everyday practice. For example, Andrea said, *“Teachers can’t expect that it will be easy and good immediately. It needs time. However, the results are worth it.”* The whole process, starting with adaptation of the next lessons, reading students’ answers, and coming back to unclear issues requires time, which can be problematic. Teachers expect help in creating FACTs because, from their point of view, it is especially time-consuming. Moreover, they agreed that all this effort is worthless without the support of the policy. Requesting deeper assessment during the educational process but ultimately focusing only on the results of the exams is very demotivating for all involved.

### Experience from FACTs Implementation

Responses in this section were more diverse (see Table 6) and represent teachers’ individual experiences. Svetlana and Maria did not adopt the following lessons based on the students’ answers, but all of the teachers discussed students’ answers, albeit with various frequencies. Similarly, Svetlana and Maria did not use FACTs outside the experimental group. Three of the teachers received feedback about using FACTs from parents.

**Table 6:**
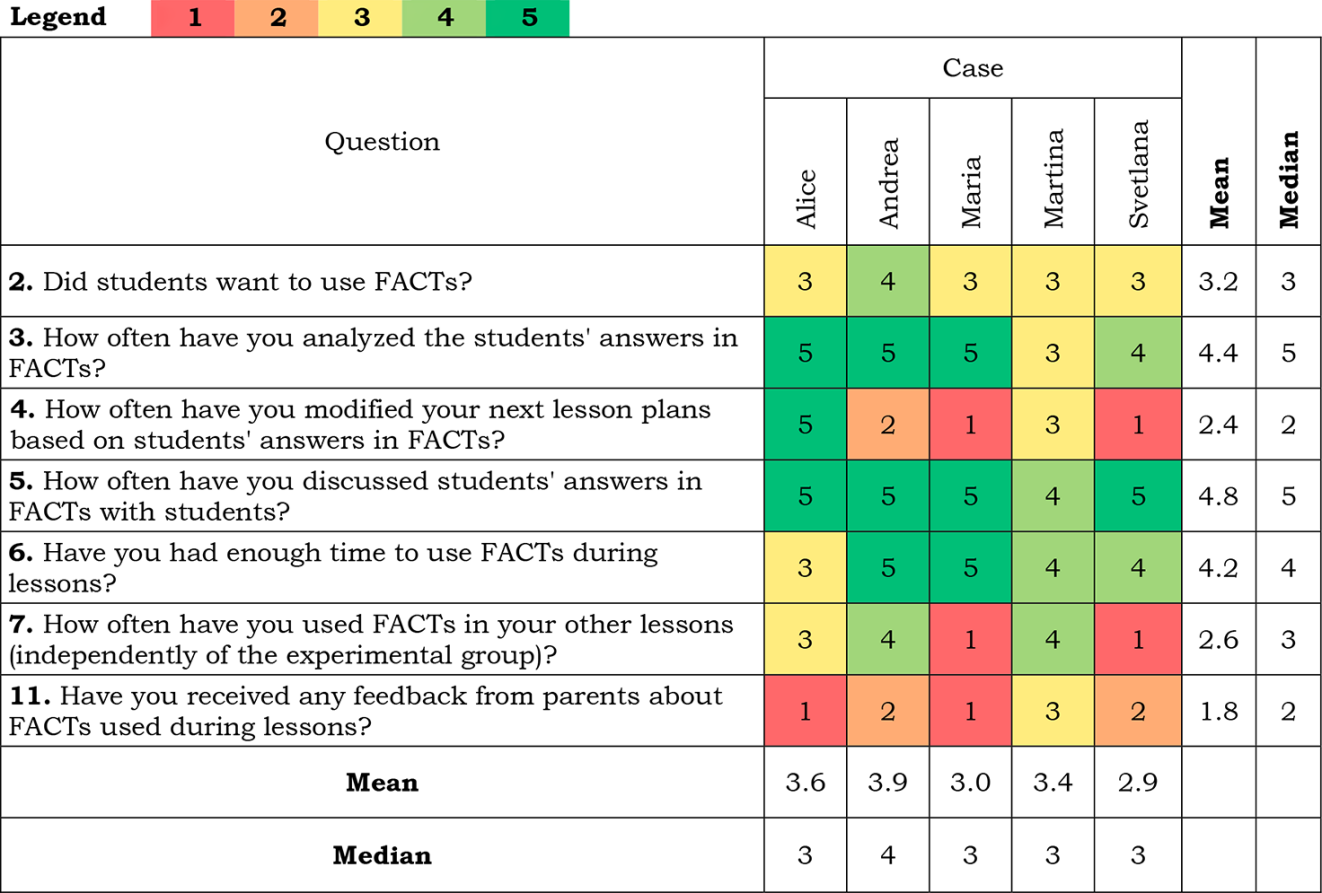
Teachers’ Answers to Questions about Experience with FA Implementation^a^

a Scale for questions 2 and 11: (1) Definitely not; (2) No; (3) It’s hard to say; (4) Yes; (5) Definitely yes; for questions 3–7: (1) Never; (2) Rarely (once or twice); (3) Occasionally (few times); (4) Often (almost after every lesson); (5) Very often (after every lesson).

### Alice

Alice tried to introduce FACTs so she could see in which way her students were reasoning. She preferred using simpler FACTs as a checklist without a descriptive part because analysis of those was simpler for her, and she was analyzing students’ answers every time. She did not use FACTs during the lesson when she taught the topic but during the next lesson. *“I wanted to give them time to work over the material at home. Just after a lesson they may not understand and remember everything, but after processing a topic at home they should master it, otherwise, there is a problem and we need to solve it.”* Alice was collecting students’ answers and read them one by one, and she analyzed FACTs at home. She marked problematic tasks and discussed them during the next lesson.

Alice was worried that students’ answers in FACTs are not always honest. *“Students want a teacher to think that they know everything, even if it is not true. They answer untruthfully, overestimate their possibilities and are not self-critical. Students have to learn how to work with FACTs and get used to them.”* Moreover, time management greatly concerned Alice. She said that students need much time to perform FACTs, especially those that are open-ended. On the other hand, Alice felt slightly insecure during the implementation process. This was because she could not plan her lessons properly. Additionally, Alice felt that she has trouble with the analysis and interpretation of some FACTs, and she would like to improve in this area.

### Andrea

At the beginning of FACTs implementation, Andrea tried to motivate her students by informing them that they would try something new—self-assessment and peer-assessment—and that they would undergo a university study. Andrea also highlighted that this assessment would not be part of their grade and that the main point was to answer truthfully. Andrea used FACTs during the same lesson when she taught the topic. Sometimes students completed the FACT at the beginning of the lesson and, in other cases, during the last 5 min. Andrea analyzed FACTs in two ways. FACTs based on true/false statements were analyzed with students. Students filled the FACT with pencils first; then, they changed to a color pen and went through the statements once again with the teacher. *“Sometimes they were naïve that they know everything. Later, they realize how difficult it is to be self-critical. I did not read these FACTs later.”* Andrea read other FACTs that required deeper analysis (e.g., exit cards) during her free time. *“I have to say that it is difficult to analyze students’ answers. I would appreciate more information about the analysis during our training at the university.”* Analysis of answers helped Andrea check whether students understood the topic and whether they could move on. *“A lack of understanding the topic causes students to think that chemistry is difficult. FACTs helped me to determine how students feel leaving the lessons and adjust the next lesson according to their needs.”* At the beginning of the following lesson, Andrea was starting with a solving problem-based task based on issues causing difficulties identified by FACTs. Moreover, analysis of FACTs helped her to adjust the same lesson run in the other classes. Andrea mentioned that, after students became used to the FACTs, they *“opened their minds...Suddenly they had no problem to be more creative and explain their opinion.”* She said that it could be because students finally realized that it does not influence their grades.

### Maria

At the beginning of FACTs implementation, Maria ensured students that their responses would not be marked by grades and only the teacher would see their responses. Moreover, she tried to convince students that FACTs would help them with learning because they would see their shortcomings. As soon as she obtained results of performance comparison between the experimental (using FACTs) and control groups, she informed students about the positive impact of FACTs on their outcomes.

Maria performed the analysis of FACTs after every lesson. She was reading FACTs at home after lessons and making notes. Maria wanted to emphasize the mistakes and explain the topic better. She not only picked areas to improve but also student’s answers that stood out positively, and she complimented them during the next lesson.

Maria liked the FACTs where students wrote questions about the lesson. *“Some questions were truly interesting. These I tried to answer. In contrast, some students wrote questions that I mentioned during my lesson. That means that students didn’t pay that much attention during the lesson.”*

On the other hand, Maria was dissatisfied when students did not engage enough in the responses. *“It truly made me mad when I saw some empty rubrics. I mean, it is fine when students write a wrong answer, or inadequate question because they are learning. However, I don’t like it when they don’t even try to think.”*

Maria saw some difficulties with the self-assessment tools. Here, a teacher can compare the truthfulness of students’ answers only with the results of a test.

One problem that Maria saw with FACTs was the teamwork. During the work in a group, students wrote the same responses, and they did not try to be unique in their answers. The second problem was time management. Students needed a lot of time to write down their answers, but they improved with time. It was difficult, especially in the beginning, when it was new for students and Maria explained every FACT individually. Sometimes, Maria found it difficult that, in the FACT, she had written something she did not mention during her lesson. Then, she must skip it and choose a different FACT. This is because FACTs are prepared before the lessons, and it is not possible to change them instantly.

The greatest advantage according to Maria was that FACTs can help students distinguish the strengths and weaknesses in their knowledge. When students identify their weaknesses, they could look at them once again and improve.

### Martina

Martina had been using elements of FA in her classroom before the training but in a simplified form (e.g., smiley face self-assessment). At the beginning of FACTs implementation, she informed students that this assessment would help them to check themselves and that it was not going to be easy at the beginning, but they would get used to it. She also decided not to mix various types of FACTs at the beginning but rather to introduce them one at a time. Students in her class were resisting FACTs at the beginning, then got used to it, and treated it as a normal part of the lesson. Nevertheless, according to Martina, 10% of the students provided very short answers. Martina tried to stimulate them by mentioning that she was reading their work.

The analysis of FACTs was difficult for Martina due to time management. She intended to analyze FACTs after each lesson, but she did not manage to do so. However, she tried to analyze it every second lesson. Sometimes they did the analysis together with the whole class immediately after filling it in. They corrected answers with different colored pens so that their shortcomings were more visible. Martina also highlighted the fact that, after each topic, she went through all FACTs once again as a type of review for the following test. *“For the test preparation, true or false statements and the Frayer model were very helpful, but the 3-2-1 card was not useful for the test preparation.”*

Martina included students’ answers in FACTs in their semester grades. *“Students wanted to know what would happen if they did not answer in FACTs. Our students are like this. I mentioned that it was going to be part of their semester assessment. However, I did it positively. I mean, when their grade was in the middle and their answers in FACTs were comprehensive, then I improved their grade. I did not assess the correctness of their answers but their diligence or effort. After the semester, we went through all FACTs once again, and they saw their progress.”* Martina mentioned that this is very motivating for students with lower science reasoning skills. *“I saw their effort there. It is visible that when students try during a lesson, they do homework, do experiments and suddenly, during the test, there was a problem. Sometimes students had problems with reading comprehension, and tests are problematic for them.”*

### Svetlana

Svetlana introduced FACTs to the class as a new kind of learning. *“I said that we have to read a lot and that we have to think about what we read. They got used to it. I explained that I won’t mark it. That it’s good to know what they have learned thus far, that it is good to see whether they move somewhere or what new material they know.”* Svetlana mentioned that students had many questions at the beginning. *“They asked me why we are going to do it. This generation is not forced to create things. Thus, I told them that if they have it all, then I will give them one good grade as a motivation. ”*

Students in Svetlana’s class answered FACTs during the lessons. Then, all students took their brochures home. *“True or false statements we checked together and I saw the final status. Otherwise, we checked at the beginning of the next lesson. It is time-consuming to give them their brochures and to collect them after each lesson. I was afraid that they could lose it, but they didn’t.”* Svetlana said that she did not have time to read every FACT after each lesson. Additionally, she mentioned that she did not know what she should correct. *“The analysis was problematic for me in general. You know, some students don’t like it when you write something in their notes. It is their notebook, and they should correct it.”* Svetlana mentioned that students’ answers were not very surprising. Most of them were caused by students’ inattention or were very simple.

As we mentioned before, Svetlana assessed students’ answers in FATCs by one final grade. It was meant as a mark for students’ work during the lessons. Everybody who has done it got a good grade. She did not give them a bad one.

Svetlana appreciated the FACTs during the laboratory experiments. It was easier for her to assess students’ work in laboratories and in teams. Similarly, she highlighted that these FACTs could motivate students who do not perform very well during the test. *“Some students don’t read the question until the end, or some didn’t get a good result in tests. However, when you ask them questions, then that kid can tell what they did during the lesson or what they learned.”*

### Teachers’ Gained Skills during FACTs Implementation

Teachers’ answers to the section Teachers’ skills gained during FACTs implementation are presented in Table 7.

**Table 7:**
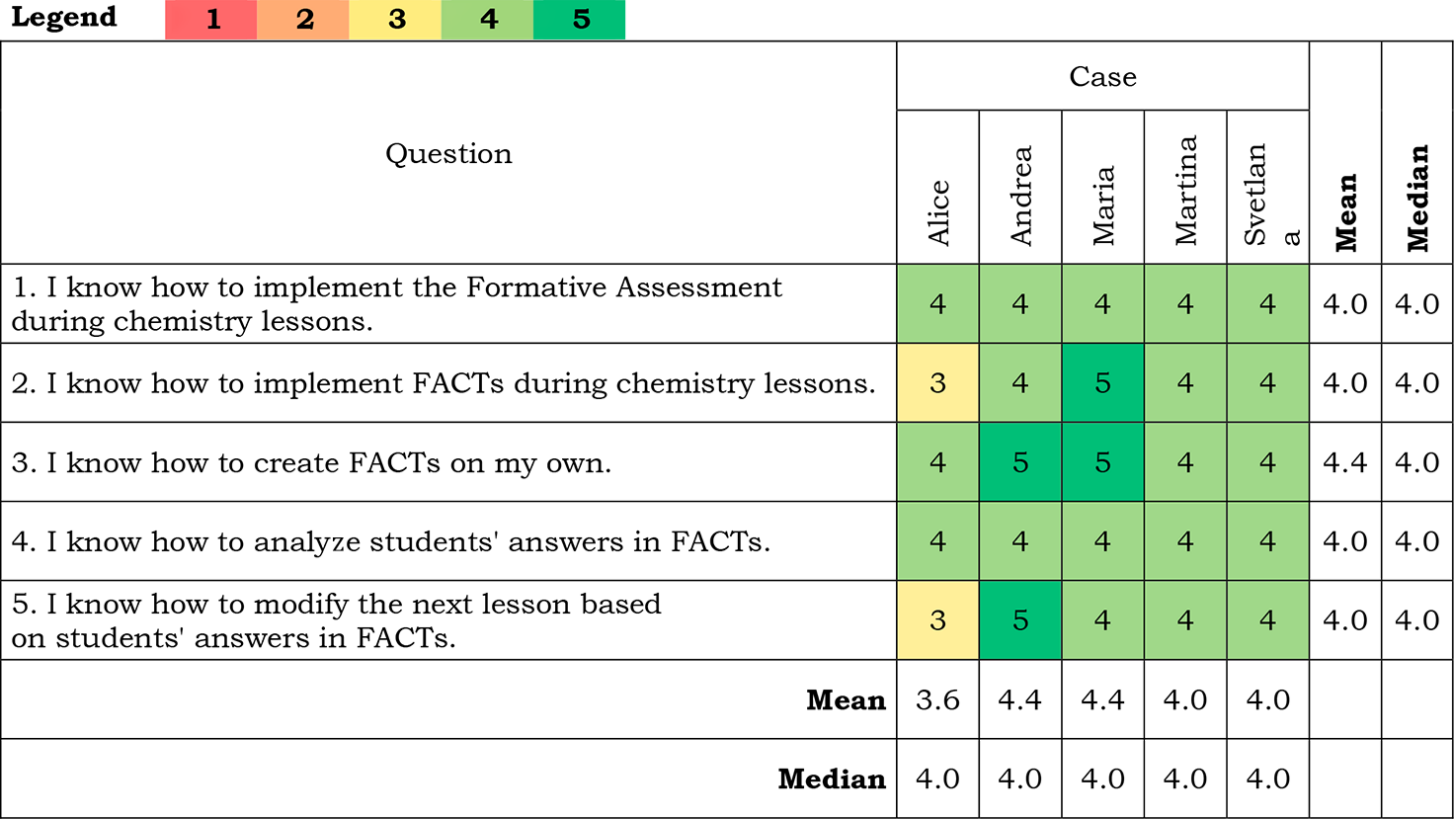
Teachers’ Answers to Questions about Their Skills^a^

a (1) Definitely not; (2) No; (3) It’s hard to say; (4) Yes; (5) Definitely yes.

In addition to the questions, Alice explained the answer “it is hard to say” in question number 2. *“I am not sure how to use every tool, e.g., vocabulary square is a little bit unclear for me.”* She again mentioned difficulties with modifying the next lesson.

Svetlana commented on her answer to the question about analyzing students’ answers in FACTs, and she said that she was not able to do it with every student individually and after every lesson.

Martina also commented on question number 5, *“You know, there are always some doubts about how to do it and whether I can do it better.”*

### Attitudes and Plans about the Future Use of FACTs

The results of this section are presented in Table 8.

**Table 8:**
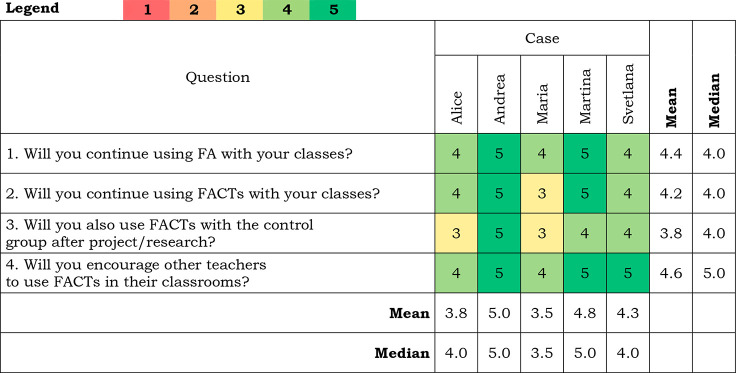
Teachers’ Answers about Their Further Use of FACTs^a^

a (1) Definitely not; (2) No; (3) It’s hard to say; (4) Yes; (5) Definitely yes.

Questions 2 and 4 required justification of the answer. The second question—Will you continue using FACTs with your classes?—had various answers from all teachers. Alice said that FACTs require a type of skill from students: *“With a control group it is difficult, they are not skilled with it.”* FACTs became a routine for Andrea: *“Of course, I think that now I cannot do it without FACTs.”* Maria explained that FACTs required some teacher’s time input: *“I do not have FACTs for the next lessons. I do not have time, and I am too lazy to create them. Therefore, I do not know.”* Martina, on the other hand, saw a different motivation to do it: *“I like it. Additionally, it is trending, it is very popular. We had an inspection and they appreciated it.”* Svetlana saw that the age of students can be a problem too, *“I have not tried it with older students.”*

For question number 4—Will you encourage other teachers to use FACTs in their classrooms?—the teachers agreed that they would. On the other hand, they emphasized that further help is needed for both pedagogy and policy.

## Discussion

### Differences during the Implementation

Using formative assessment or FACTs during STEM lessons, especially chemistry lessons, has its challenges, and every teacher addresses them in his or her own way.^74^ The results from our questionnaire about teachers’ experience with FACTS illustrate this very well. Alice is the most sceptical teacher, with a mean answer of 3.6 (median 4). Alice was the only teacher who did not use FACTs during the lesson when she taught the topic but rather one lesson later because she considered the learning to be a longer process. Alice reported problems with implementing FACTs, analyzing students’ answers, and adapting the following lessons. In contrast, Andrea had the most positive attitude. Her answers in the part about teachers’ beliefs had a mean of 4.6, and she was the only teacher with a median of 5. She answered without strong agreement (by 4 – “Yes”) only once (Using FACTs during chemistry lessons influenced students’ learning in other subjects). Moreover, Andrea’s self-assessment of skills was very optimistic, and she was the teacher with the most ambitious plans—all answers with 5. Andrea’s positive attitude could grow out of her prior experience since she declared that she had used FA very often before the training. She mentioned it in one of her answers, *“Teachers can’t expect that it will be easy and good immediately. It needs time. However, the results are worth it.”* Sach^75^ put in relation to teachers’ perceptions of formative assessment and the length of teachers’ experience. As Sach describes, teachers’ confidence can grow with their experience. Despite her optimism and practice, Andrea mentioned problems with the analysis of students’ answers. Svetlana declared the least experience with FA before the training. Nevertheless, her beliefs, gained skills, and plans are positive. For Svetlana, the biggest concern was time management; she also had doubts about what age was appropriate for FACTs usage: *“I have not tried it with older students.”* Maria was using FACTs before the training occasionally. She felt very confident about her ability to use FACTs during chemistry lessons; however, she did not avoid problems with the implementation. Maria said that sometimes she could not use FACTs because there were things mentioned that she did not manage to discuss with students during the lesson. She also pointed out the problem with the standardization of FACTs. Moreover, she is not sure whether she will continue to use FACTs. *“I would use it if I had it prepared. However, I don’t have time to prepare it on my own.”* As Wiliam^76^ reflects, in-service support for teachers is necessary to help them develop teaching approaches. Wiliam claimed that this is not the only factor but combined with other issues, such as class-size reduction, the results might be different. Martina did not have many problems with using FACTs. Time management was the one issue she pointed out. However, Martina sees a large advantage of FACTs for students with low scientific reasoning skills. She also had an external motivation to use it: *“Additionally, it is modern and it is very popular. We had an inspection and they appreciated it.”*

### Involving and Motivating Students: Are They Being Self-critical?

It is important to know how to introduce a formative assessment to students who will be using it during lessons. Moreover, students must understand the meaning and effect of offered feedback.^77^ However, this can be very difficult when the national system supports only the summative interpretation of students’ performance.^65^ As the obtained results indicate, teachers struggle with involving students in the process. In the beginning, explaining the importance of formative assessment and motivating students to engage might be challenging. Alice explained it very well: *“In general, society requires results. They do not care about the process. They need percentages, grades, and numbers. It is difficult to work with students, ask them to explain stuff when finally, after the whole study they just need a number.”* At the same time, it is difficult for students to start being self-critical and speak honestly about their skills, understanding, and knowledge. Alice described it as follows: *“Students want a teacher to think that they know everything even if it is not true. They answer untruthfully, they overestimate their possibilities, and they are not self-critical. Students need to learn how to work with FACTs.”* Formative assessment and giving feedback to students do not necessarily or “magically” improve students’ performance without their actions.^78^ However, Andrea pointed out, *“When students saw it is not marked, they improved in self-assessment, they started to answer more critically, but what is more important, they started to answer truthfully.”* We still lack information on how to help students deliver the feedback and, at the same time, motivate them to perform during formative assessment and to be self-critical. Teachers reported problems with teamwork. Teachers said that students tend to write the same answers and that they are not creative. Sridharan, Tai, and Boud^79^ also identified problems with formative assessment and teamwork. They reported problems with students’ honesty during peer assessment and teamwork, especially when it is related to summative assessment. These concerns were also reported in studies at the higher education level.^80−82^ However, Andrea also observed, *“Students knew that they cannot be passive because they will receive a bad peer assessment.”*

### Every Student Matters

As the use of FACTs in the classroom continued, teachers in our study reported an increase in students’ engagement. Svetlana made an interesting statement: *“They didn’t talk about some other things or the following lessons. They discussed their questions and possible answers.”* Using FACTs offers an opportunity to involve every student, and it encourages students who would not otherwise find the courage to ask or discuss issues. A similar effect can be achieved using an inquiry-based learning strategy; however, this approach requires more time and work with smaller groups.^83^ Based on students’ ideas, answers, and questions, teachers should reflect and adapt the following lessons. Martina said that this was the biggest change for her: *“Sometimes we just go through the syllabus and now I am curious about students’ questions and their opinions. With FACTs, even shy students can write their opinions, and I can read them.”*

### When the Time Is Running out

There are also elements of FACTs that are clearly problematic for teachers, that is, answers’ analysis and lessons adaptation according to FA results. Teachers mentioned problems with analysis, especially with some particular FACTs, such as the Frayer model. In the result, they chose those that were easier for the analysis. The analysis of students’ answers and adapting subsequent lessons are vital parts of formative assessment and, at the same time, are very problematic. For both, timing is crucial. Otnes and Solheim^84^ mentioned that timing is essential for *“when”* the feedback is given but similarly for the time allocated for instruction. Svetlana mentioned, *“I could have read it more often, but there was no time for it. I regret it now.”* Digital technologies seem to be a solution since teachers can adjust the questions, organize answers, or group them.^85−88^ However, it should be kept in mind that teachers should focus on pedagogy and teaching rather than technology.^87^

### How about Involving Parents?

As formative assessment develops, it offers new opportunities and dilemmas. Involving parents in the process is one of them. The thing our teachers agree on is that FA must be conducted in a meaningful way. Engaging parents too much or too soon could be counterproductive. Alice commented, *“When a student cannot write a definition of something, how can he use that term in more difficult operations such as analysis? However, it is difficult because parents could see it as more work for them.”* It is common for parents to be included in children’s preparations for summative assessment, but mostly, the grade is the only result parents see from their children’s performance. Martina received feedback from one of her students’ parents. *“He didn’t perform very well in the test, and his father came to talk with me. When I showed him the FACTs and student’s answers, the father agreed on how we work during lessons together.”* Andrea also commented, *“When parents came to discuss the kid’s performance, the FACTs were a useful tool to support my arguments. When parents saw kid’s answer: ‘I can do it only with my teacher’s help’, good performance could hardly be expected.”* As teachers suggested, engaging parents may be done after the introductory period. Dagdag and Dagdag^89^ informed parents quarterly. As emphasized, parents may play a key role in students’ learning. Parents can help to build the children’s strengths and address their weaknesses.^89,90^ However, involving parents in formative assessment may also help students with special needs or disabilities. There are studies on engaging parents of disabled students in formative assessment implementation, but more research needs to be done in this area.^91−93^

## Conclusions

Educational systems at the secondary level in many countries are dominated by summative assessment and focused on the preparation of students for exams rather than solving problems, critical thinking, and applying scientific knowledge in everyday life. This also applies to chemistry education. The general trend of shifting toward meaningful learning with a focus on the process of learning is invisible. In such reflective education, formative assessment plays an important role. Even though this kind of assessment has been known for decades, it is still not commonly used in secondary chemistry education. Moreover, there are still debates about how FA should be realized, especially with limited lesson time, with the acceptable efforts of teachers, and with taking into account the specifics of chemistry lessons (many abstract information, calculations, or laboratory experiments). Previous results suggest that FACTs can be considered an adequate tool, but there is still limited information on how they work in chemistry lessons at the secondary school level. Therefore, this research study focuses on teachers’ experiences, practices, and attitudes about the implementation of formative assessment based on FACTs during chemistry lessons. The results suggest that FACTs are a great tool to recognize students’ strengths and weaknesses, and such information is available not only for teachers but also for students themselves to guide them through the learning process, highlighting the key points of the topic. This is especially helpful when new chemistry topics with a lot of information are introduced. Moreover, FACTs deliver information on students’ performance in these areas, which helps them steer their learning, prepare for summative assessment, and take responsibility for their learning. FACTs are based on students’ peer and self-assessments and, therefore, rely on students’ truthfulness. Students need to perceive this kind of assessment as a feedback tool rather than an assessment tool. They need to realize that it serves them, not the teacher. This way of communication may help students not only during chemistry education but also during life, and they can use it during their team work or job communication or when receiving feedback in general. On the other hand, FACTs serve and help teachers track students’ progress and identify and solve problems immediately during and/or after the lesson. In chemistry education, a lot of information is connected to each other and not understanding one principle may lead to misunderstanding the whole topic. In summative assessments, teachers receive such information after the test. At that point, going back to the topic may be not possible, for example, due to time limitations. In classes with many students, FACTs provide an opportunity for every student to present and explain its status in a relatively short time. Moreover, it offers a place to ask questions that imitates chemistry inquiry where research questions need to be asked. As we see, this kind of assessment opens minds and provides a time and place for reflection. Teachers note the high potential of FACTs in supporting students’ practical work (e.g., during laboratory experiments); they see the value of personalized FACTs analysis elaborated in detail with each student and that assessing students in a formative way may move them forward more than a traditional assessment by a grade.

Despite the benefits, there are problematic aspects of FACTs implementation that require further research, clarification of the FACTs methodology, and teacher training. First, the preparation of FACTs is considered difficult. Additionally, preparing extra tools is time-consuming especially when teachers must normally prepare experiment demonstrations or laboratories on their own. Teachers require assistance in FACTs implementation, especially at the beginning of the process, and they expect various examples of best practices of the FACTs application supported by evidence of their positive impact. Another challenging area is FACTs analysis. Both, the time required for reading students’ answers and the analysis of the content are problematic. Examples of how to analyze students’ answers are needed, especially for open-ended techniques. Moreover, teachers described the lack of unique answers during teamwork. Teachers are also afraid of adapting lessons that follow FACTs. They see that it can disrupt their plans, especially if they do not follow the lesson scenarios or when a laboratory lesson follows. The process of FACTs implementation requires flexibility in planning lessons ahead. Teachers need to adapt lessons according to students’ answers. Similarly, FACTs need to be changed appropriately.

The policy plays a significant role in formative assessment implementation. Teachers believe that formalization of FA at the school level would simplify the process and would have a tremendous impact on teachers’ classroom practice and students’ attitudes. Using FACTs during science lessons would help teachers to implement them and students to recognize them. Moreover, relations between summative and formative assessment should be established, but in this case, possible rules and dependencies require deeper research and analysis.

Furthermore, involving parents in the whole process offers a new perspective and may play a crucial role in students’ engagement in learning chemistry. Students and parents may work on particular goals instead of focusing on general topics. On the other hand, FACTs may help teachers justify students’ poor performance. In contrast, students with special needs may also benefit from this assessment since their shortcomings would be more personalized. Additionally, it might be possible to provide these students not only with information about their shortcomings but also with the possible reasons for and detailed descriptions of these gaps.

## Limitations and Implications

To generalize and interpret the results of the present study, we should note its limitations. First, participating teachers were recruited in one country (Slovakia); therefore, local implications can have a significant influence on the results. All the teachers participated in the training and research voluntarily, so their attitude toward innovations and presented methods could be more positive than average. Moreover, all participating teachers were women. A higher number of cases and better diversity would provide a more representative view of the studied effects. Furthermore, the training and the research were conducted by the same educators; therefore, judgment about teachers’ actions could be subjective to some extent. Additionally, the research is based on teachers’ relations and interpretations. Monitoring the process and collecting evidence (as in the first stage of implementation and the research) could increase the objectivity of the results, although it may influence teachers’ behavior.

Despite these shortcomings, the findings presented in this research may be interesting and useful for chemistry educators designing and running professional development programs for teachers in the FA area. Educators should keep in mind that, during the training, teachers need to see the advantages of the presented method, preferably with research-based evidence gathered in their classroom. The results showed that FACTs are a very powerful and useful FA technique that can be successfully used with secondary school students during chemistry (science) lessons. However, its implementation is a long process during which teachers require assistance. The implementation must be flexible, and teachers must be in charge, especially in the later phase of the process. During the training, educators should focus on FACTs results’ analysis, preferably with practical, real classroom examples. Additionally, educators must be reassured about the clarity of every FACT,; otherwise, teachers would not use them. During the implementation, teachers may also expect assistance with the development of the FACTs. A bank of FACTs could be very useful to achieve this. It is easier for teachers to design new FACTs basing on existing examples that can be adapted.

Considering the FACTs implementation process, teachers advise starting with younger students. Chemistry teachers should start with easier topics to introduce FACTs to students. Similarly, implementing FACTs in more subjects may simplify the whole process.

Further research could be focused on simplifying the FACTs implementation process, analyzing students’ answers, involving parents in the assessment process, and helping students with special needs.

## Supporting Information Available

Questionnaire (PDF; DOCX)
