# Supplementary material for: Introduction of Formative Assessment Classroom Techniques (FACTs) to School Chemistry Teaching: Teachers’ Attitudes, Beliefs, and Experiences
Source: J Chem Educ. 2023 Aug 25;100(9):3276–90. doi: 10.1021/acs.jchemed.3c00591 (PMC10501440; doi:10.1021/acs.jchemed.3c00591)
Supplement: Supplementary file 1 — ed3c00591_si_001.pdf [file ed3c00591_si_001.pdf]

Supporting Information

for

Introduction of Formative Assessment Classroom Techniques (FACTs) to School Chemistry Teaching: Teachers' Attitudes, Beliefs, and Experiences

Mária Babinčáková*[a,c], Mária Ganajová[b], Pawel Bernard[c]

[a]Pavol Jozef Šafárik University in Košice, Lifelong Learning Centre and Project Support, Šrobárova 2, 041 80, Košice, Slovakia

[b]Pavol Jozef Šafárik University in Košice, Faculty of Science, Department of Didactics of Chemistry, Šrobárova 2, 041 80, Košice, Slovakia

[c] Jagiellonian University, Faculty of Chemistry, Department of Chemical Education, Gronostajowa Str.2, Kraków, 30-387, Poland

*Corresponding author: maria.babincakova@uj.edu.pl

Supporting Information

Questionnaire: Chemistry Teachers' Views on Teaching with FACTs

Name: School: Date:

Questionnaire: Chemistry Teachers' Views on Teaching with FACTs

Previous use of formative assessment

1. **Can you define FA? You can use your own words.** (open question)

2. **Did you know this definition before the training?**

1. YES 2. NO

A. **<u>If Yes:</u> Where have you learned this definition?** (open question)

B. **How often have you used FA before the training and research?**

1. Never	2. Rarely (few times a year)	3. Occasionally (about once a month)	4. Often (few times a month)	5. Very often (almost every lesson)

a) **<u>If Yes</u>: How often have you used FACTs before the training and research?**

1. Never	2. Rarely (few times a year)	3. Occasionally (about once a month)	4. Often (few times a month)	5. Very often (almost every lesson)

b) **<u>If No: Do you know other types of assessment?</u>**

1. YES 2. NO

a. **<u>If Yes</u>: Can you define it?** (open question)

Experience form FA implementation

1. **How did you introduce FACTs to the students?** (open question)

2. **Did students want to use FACTs?**

1. Definitely not 2. No 3. It's hard to say 4. Yes 5. Definitely yes

   A. **<u>If No:</u> How did you solve this problem?** (open question)

3. **How often have you analysed the students' answers in FACTs?**

1. Never	2. Rarely (once or twice)	3. Occasionally (few times)	4. Often (<u>almost</u> after every lesson)	5. Very often (after every lesson)

   A. **Can you tell me why you did it so?** (open question)

   B. **<u>If Yes:</u> How have you analysed them?** (open question)

   C. **<u>If Yes:</u> Have you experienced any problems with analysing students' answers in FACTs?**

1. Never	2. Rarely (once or twice)	3. Occasionally (few times)	4. Often (<u>almost</u> every time)	5. Very often (every time)

a) **<u>If Yes:</u> What kind of problems have you experienced?** (open question)

4. **How often have you modified your next lesson plans based on students' answers in FACTs?**

1. Never	2. Rarely (once or twice)	3. Occasionally (few times)	4. Often (<u>almost</u> after every lesson)	5. Very often (after every lesson)

A. **Why have you decided to (not) modify them?** (open question)

B. **<u>If Yes:</u> Which parts of the lesson have you modified?** (open question)

C. **<u>If Yes:</u> What kind of changes have you incorporated?** (open question)

D. **<u>If Yes:</u> Have you experienced any problems with the modification of your next lesson plans?**

1. Definitely not	2. No	3. It's hard to say	4. Yes	5. Definitely yes

a) **<u>If Yes:</u> What kind of problems have you experienced?** (open question)

E. <u>If Yes:</u> Is modifying lesson plans basing on students' answers in FACTs something that a teacher should always do?

1. Definitely not 2. No 3. It's hard to say 4. Yes 5. Definitely yes

5. How often have you discussed students' answers in FACTs with students?

1. Never
2. Rarely (once or twice)
3. Occasionally (few times)
4. Often (almost every time when I used FACTs)
5. Very often (every time when I used FACTs)

A. <u>If Yes:</u> How often have you used students' answers in FACTs to define a problematic task?

1. Never
2. Rarely (once or twice)
3. Occasionally (few times)
4. Often (almost every time when I used FACTs)
5. Very often (every time when I used FACTs)

B. <u>If Yes:</u> Have you had any problems to choose the problematic tasks from students' answers in FACTs? (open question)

C. <u>If Yes:</u> How often have you discussed student's answers in FACTs individually?

1. Never
2. Rarely (once or twice)
3. Occasionally (few times)
4. Often (almost every time when I used FACTs)
5. Very often (every time when I used FACTs)

D. <u>If Yes:</u> How often have you discussed student's answers in FACTs with the entire class?

1. Never
2. Rarely (once or twice)
3. Occasionally (few times)
4. Often (almost every time when I used FACTs)
5. Very often (every time when I used FACTs)

6. Have you had enough time to use FACTs during lessons?

1. Never
2. Rarely (once or twice)
3. Occasionally (few times)
4. Often (almost every time when I used FACTs)
5. Very often (every time when I used FACTs)

7. How often have you used FACTs in your other lessons (independently of the experimental group)?

1. Never
2. Rarely (few times)
3. Occasionally (about once a month)
4. Often (few times a month)
5. Very often (almost every lesson)

8. Have you used students' answers in FACTs for their semester mark?

1. YES 2. NO

A. Why have you (not) done that? (open question)

B. <u>If Yes:</u> How have you done that? (open question)

C. It is good to use students' answers in FACTs for their semester mark

1. Definitely not 2. No 3. It's hard to say 4. Yes 5. Definitely yes

c) **Why do you think so?** (open question)

9. What kind of materials do you expect from trainers/educators? (multiple choice)

a) general ideas for FA
b) the whole lessons' scenarios with FACTs embedded
c) particular FACTs without the scenario
d) exemplary FACTs with the scenario
e) exemplary FACTs without the scenario
f) nothing

A. Can you justify your answer? (open question)

10. Did you experience any problems using FACTs generally? (open question)

11. Have you received any feedback from parents about FACTs used during lessons?

1. Never	2. Rarely<br>(once or twice)	3. Occasionally<br>(few times)	4. Often<br>(almost every time when I used FACTs)	5. Very often<br>(every time when I used FACTs)

A. What kind of feedback? (open question)

12. What was the most significant change in your teaching as a result of participating in the training and this study? (open question)

Questions – Teacher's beliefs

Do you agree with the claims presented below?

1. Using FACTs has a positive impact on the atmosphere during chemistry lessons.

1. Definitely not 2. No 3. It's hard to say 4. Yes 5. Definitely yes

2. Using FACTs improves the quality of students' learning generally.

1. Definitely not 2. No 3. It's hard to say 4. Yes 5. Definitely yes

A. Why do you think so? (Open question)

3. Using FACTs raises students' interest in learning.

1. Definitely not 2. No 3. It's hard to say 4. Yes 5. Definitely yes

4. Using FACTs improves students' confidence in learning.

1. Definitely not 2. No 3. It's hard to say 4. Yes 5. Definitely yes

5. Using FACTs encourages students to work harder.

1. Definitely not 2. No 3. It's hard to say 4. Yes 5. Definitely yes

6. Using FACTs encourages autonomous learning of students.

1. Definitely not 2. No 3. It’s hard to say 4. Yes 5. Definitely yes

7. Using FACTs helps students to recognize their strengths and weaknesses in their knowledge.

1. Definitely not 2. No 3. It’s hard to say 4. Yes 5. Definitely yes

8. Using FACTs helps me to identify students' strengths and weaknesses in their knowledge.

1. Definitely not 2. No 3. It’s hard to say 4. Yes 5. Definitely yes

9. Using FACTs during chemistry lessons influenced students' learning in other subjects.

1. Definitely not 2. No 3. It’s hard to say 4. Yes 5. Definitely yes

10. Using FACTs improves the quality of teaching generally.

1. Definitely not 2. No 3. It’s hard to say 4. Yes 5. Definitely yes

A. Can you justify your answer? (open question)

11. Using FACTs improves the quality of my teaching.

1. Definitely not 2. No 3. It’s hard to say 4. Yes 5. Definitely yes

A. Can you justify your answer? (open question)

12. Do you think that parents should be informed about the answers in FACTs of their children?

1. Definitely not 2. No 3. It’s hard to say 4. Yes 5. Definitely yes

B. Can you justify your answer? (open question)

13. Would you recommend using FACTs to other teachers?

1. Definitely not 2. No 3. It’s hard to say 4. Yes 5. Definitely yes

A. <u>If Not:</u> Can you justify your answer? (open question)

B. <u>If Yes:</u> What would you say them? (open question)

C. <u>If Yes: How often would you recommend that they use FACTs?</u>

1. Never	2. Rarely (few times a year)	3. Occasionally (about once a month)	4. Often (few times a month)	5. Very often (almost every lesson)

Skills gained during training

1. I know how to implement the Formative Assessment during chemistry lessons.

1. Definitely not 2. No 3. It's hard to say 4. Yes 5. Definitely yes

2. I know how to implement FACTs during chemistry lessons.

1. Definitely not 2. No 3. It's hard to say 4. Yes 5. Definitely yes

3. I know how to create FACTs on my own.

1. Definitely not 2. No 3. It's hard to say 4. Yes 5. Definitely yes

4. I know how to analyse students' answers in FACTs.

1. Definitely not 2. No 3. It's hard to say 4. Yes 5. Definitely yes

5. I know how to modify the next lesson basing on students' answers in FACTs.

1. Definitely not 2. No 3. It's hard to say 4. Yes 5. Definitely yes

Plans

1. Will you continue using FA with your classes?

1. Definitely not 2. No 3. It's hard to say 4. Yes 5. Definitely yes

2. Will you continue using FACTs with your classes?

1. Definitely not 2. No 3. It's hard to say 4. Yes 5. Definitely yes

A. Can you justify your answer? (open question)

3. Will you also use FACTs with the control group after project/research?

1. Definitely not 2. No 3. It's hard to say 4. Yes 5. Definitely yes

A. Can you justify your answer? (open question)

4. Will you encourage other teachers to use FACTs in their classrooms?

1. Definitely not 2. No 3. It's hard to say 4. Yes 5. Definitely yes

A. Can you justify your answer? (open question)
